# Clinical Development of New TB Vaccines: Recent Advances and Next Steps

**DOI:** 10.3389/fmicb.2019.03154

**Published:** 2020-01-30

**Authors:** Mark Hatherill, Richard G. White, Thomas R. Hawn

**Affiliations:** ^1^South African Tuberculosis Vaccine Initiative (SATVI), Division of Immunology, Department of Pathology, Institute of Infectious Disease & Molecular Medicine, University of Cape Town, Cape Town, South Africa; ^2^TB Modelling Group, TB Centre – Centre for the Mathematical Modelling of Infectious Diseases, Faculty of Epidemiology and Population Health, London School of Hygiene & Tropical Medicine, London, United Kingdom; ^3^Division of Allergy and Infectious Diseases, Department of Medicine, University of Washington, Seattle, WA, United States

**Keywords:** vaccine, development, Bacille Calmette Guerin, *Mycobacterium tuberculosis*, tuberculosis

## Abstract

*Mycobacterium tuberculosis* (Mtb) kills more people worldwide than any single infectious pathogen, yet the only vaccine licensed against tuberculosis, Bacille Calmette Guerin (BCG) is approaching its centenary. Two recent advances in clinical tuberculosis vaccine development have invigorated the field. BCG revaccination of interferon-gamma release assay (IGRA) negative adolescents provided 45% protection against sustained Mtb infection defined by IGRA conversion; and the protein-subunit vaccine M72/AS01_*E*_ provided 50% protection against progression from Mtb infection to tuberculosis disease in IGRA-positive adults. These findings provide encouraging evidence for pre-exposure and post-exposure approaches to vaccination against tuberculosis, both of which may be necessary to rapidly interrupt the cycle of Mtb transmission and sustain long-term impact on global tuberculosis control. New trials are needed to demonstrate efficacy of M72/AS01_*E*_ with greater precision, in a wider age range, in diverse epidemic settings, and in populations that include Mtb-uninfected and HIV-infected persons. Modeling the impact of mass campaigns with M72/AS01_*E*_ and other fast-follower vaccine candidates will be crucial to make the use case and demonstrate public health value for TB endemic countries. The size and scope of the next generation of efficacy trials, and the need to expand and accelerate the existing clinical development pipeline, will require public and private consortium funding and concerted political will.

## Introduction

The global tuberculosis (TB) epidemic is a chronic humanitarian tragedy that continues unchecked despite, or perhaps because of the fact that the only vaccine licensed against TB, the world’s leading cause of death by an infectious pathogen (World Health Organizataion [WHO], 2018c), has been used for almost a century. Infant Bacille Calmette Guerin (BCG) vaccination provides consistent protection against the most severe forms of childhood TB, such as miliary and meningeal TB disease (Mangtani et al., 2014). However, with the possible exception of low TB incidence settings (Aronson et al., 2004; Barreto et al., 2005; Abubakar et al., 2013; Mangtani et al., 2017), BCG efficacy wanes in adolescence (Sterne et al., 1998). BCG vaccination offers little or no protection against adult-type pulmonary TB (Mangtani et al., 2014), which is responsible for the transmission of *Mycobacterium tuberculosis* (Mtb) to susceptible people. It is thus not surprising that despite universal infant BCG vaccination in TB-endemic countries, an estimated 10 million people developed TB and 1.6 million people died from the disease worldwide in 2017, including 300,000 HIV/TB co-infected patients (World Health Organizataion [WHO], 2018c). Given the scale of the global TB epidemic and the slow pace of TB control efforts, it is clear that a new and more effective TB vaccine is needed to achieve World Health Organization (WHO) End TB Strategy targets by reducing TB deaths by 95% and new cases of TB by 90% by 2035 (Abu-Raddad et al., 2009; Uplekar et al., 2015).

## Target Populations for a New Tuberculosis Vaccine

The WHO has developed Preferred Product Characteristics (PPC) (Schrager et al., 2018) and identified two target populations for new TB vaccines: (1) adolescents and adults; and (2) infants. It is recognized that although a new infant TB vaccine that offers more effective durable protection than BCG would be desirable, any new infant vaccination strategy would require many years to demonstrate major impact on the epidemic (Knight et al., 2014). For example, mass vaccination of adults using a new TB vaccine with only 40% efficacy would achieve the same initial reduction in TB incidence 20 years earlier than an infant vaccine with 80% efficacy and lifelong protection (Knight et al., 2014). Developing a new vaccine for use in mass campaigns to prevent pulmonary TB among adolescents and adults will be necessary to interrupt the cycle of Mtb transmission in the short- and medium-term (Knight et al., 2014), whereas some combination of infant and adolescent/adult vaccination approaches would likely be optimal to sustain long-term TB control (Harris et al., 2016).

Approximately 23% of the global population is thought to be infected with Mtb (Houben and Dodd, 2016), forming a large reservoir of future TB cases that includes both drug-sensitive (DS) and drug-resistant (DR) TB strains. However, it is recognized that individuals with long-standing quiescent Mtb infection that has historically been termed “latent” are relatively protected, with 79% lower risk against progression to TB disease in the face of reinfection than uninfected people (Andrews et al., 2012). It has been proposed that many people thought to have long-standing “latent” infection, although sensitized to Mtb, are predominantly uninfected and at much lower risk of subsequent disease (Behr et al., 2018). Although only 10–15% of Mtb-infected people will progress to TB disease in their lifetime, risk of progression is most likely in the 1–2 years immediately after initial Mtb infection (Behr et al., 2018). Therefore, the uninfected 77% of the global population (approximately 6 billion people) form a large pool of susceptibles amongst whom, once exposed, those who become newly infected would be at highest risk of developing TB disease. This paradox, the large number of chronically Mtb-infected people at long-term low risk, and the much smaller number of recently infected and “yet-to-be-infected” people at short-term high risk, poses a major challenge for TB vaccine development in identifying who should be vaccinated and when. That challenge is compounded by our inability to precisely define the time of Mtb exposure and the lack of direct tests for infection. Current tests for immune sensitization to Mtb, such as the interferon-gamma release assay (IGRA), cannot differentiate between distant and recent infection. Therefore, those recently Mtb-infected individuals at highest risk of progression to TB, estimated at 0.8% of the global population (55 million people), largely remain hidden among the much larger pool of 1.7 billion “latently” infected people (Houben and Dodd, 2016).

How can a new TB vaccination strategy offer protection both to “most of those at risk,” as well as “those most at risk,” given current limitations of predictive and diagnostic tests? Large cohort studies have identified proteomic (Penn-Nicholson et al., 2019) and transcriptomic (Suliman et al., 2018b; Zak et al., 2016; Warsinske et al., 2018) host blood biomarkers and high IGRA conversion threshold values (interferon-gamma >4.0 IU/mL) (Winje et al., 2018; Andrews et al., 2017) that predict which infected individuals have highest risk of progression to TB disease. However, it is highly unlikely that pre-vaccination screening, even to identify IGRA-positive people, would be feasible in high TB incidence developing countries with limited resources and constrained health system capacity.

It is likely that future TB vaccination programs would use age-targeting to tailor routine vaccination schedules and mass campaigns for either Mtb-uninfected infants, children, and pre-adolescents in a pre-exposure strategy, or for Mtb-infected adolescents and adults in a post-exposure strategy, to maximize cost-effectiveness (Harris et al., 2016). However, the feasibility and efficiency of age-targeted vaccination would likely differ by country, depending on age-specific prevalence of Mtb infection and TB disease, which varies widely even in high TB burden countries. For example, prevalence of Mtb infection defined by positive IGRA in China was 3% in adolescents, rising to 33% in older adults, consistent with the epidemiology of an aging TB epidemic (Gao et al., 2015). It follows that a post-exposure vaccination campaign for Mtb-infected people in China should target primarily older adults, an approach which has the maximum modeled population-level impact on the Chinese epidemic (Harris et al., 2019). By contrast, in South Africa, 49% of adolescents and 56% of adults were IGRA-positive (Bunyasi et al., 2019; Mahomed et al., 2006), consistent with a high rate of ongoing Mtb transmission to younger people. Notably, although a post-exposure vaccination campaign among South African adolescents would include a greater proportion of IGRA-positive individuals than in China (Gao et al., 2015; Bunyasi et al., 2019), half of adolescent vaccinees would still be Mtb-uninfected. Lower prevalence rates of Mtb infection among adolescents in e.g., Kenya (32%) (Nduba et al., 2019) and Uganda (16%) (Mumpe-Mwanja et al., 2015), would favor a pre-exposure rather than a post-exposure approach for adolescents in these and other countries with lower Mtb transmission rates (Gao et al., 2015). This distinction is important, because TB disease incidence rises sharply from 15 to 25 years of age in endemic countries (Hermans et al., 2015). The benefit of delay in vaccination until adulthood to allow for higher prevalence of Mtb infection in a post-exposure vaccination strategy, would be counter-balanced by missed opportunities to prevent TB cases among younger people.

## Pre-Exposure Approaches to Infant Vaccination

The principle that an individual should be vaccinated before, rather than during or after the period of highest risk for disease (Behr et al., 2018), suggests that pre-exposure vaccination of Mtb-uninfected people might eventually have greater population-level impact than post-exposure vaccination of Mtb-infected people. This approach hinges on the key assumptions that pre-exposure vaccination is feasible and, crucially, offers durable long-term protection lasting decades (Schrager et al., 2018) Figure 1.

**FIGURE 1:**
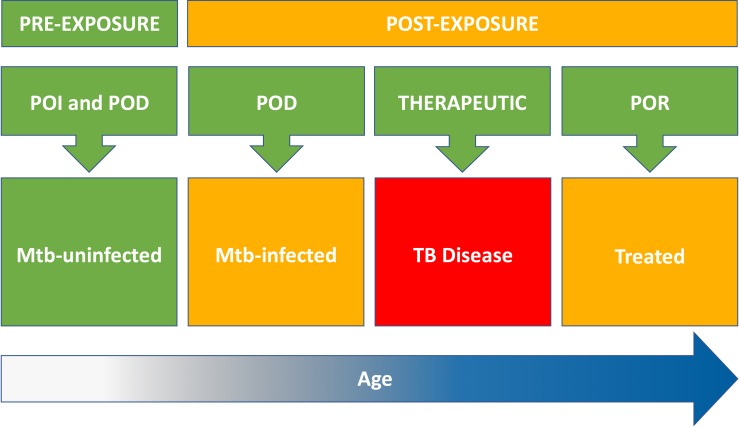
Target populations for new TB vaccines include Mtb-uninfected (pre-exposure) and Mtb-infected, TB diseased, and treated individuals (post-exposure). Risk of Mtb infection and progression to TB disease increases with age (time at risk). Pre-exposure strategies for TB vaccination of Mtb-uninfected individuals include both prevention of infection (POI) and prevention of disease (POD). Post-exposure strategies include vaccination of Mtb-infected individuals to prevent progression to TB disease (POD); vaccination of TB patients to improve treatment outcomes (therapeutic); and vaccination of treated TB patients to prevent recurrent disease (POR). Therapeutic vaccination might also confer POR benefit.

Can pre-exposure vaccination of Mtb-uninfected humans protect against subsequent TB disease? The clearest evidence is the example of BCG vaccination for newborn infants, the classical Mtb-unexposed and Mtb-uninfected population. BCG vaccination in infancy has approximately 59% efficacy against childhood pulmonary TB (Mangtani et al., 2014), but offers 90% protection against severe childhood disease phenotypes such as miliary and meningitic TB (Mangtani et al., 2014), which are largely responsible for the high TB mortality in this age group. The possibility that infant BCG vaccination also confers non-specific benefits, including reduction in early all-cause mortality, remains controversial (Aaby et al., 2011; Fine et al., 2012). The efficacy of infant BCG vaccination against childhood TB, demonstrated in many observational studies and randomized trials since BCG was first used in 1921, has resulted in universal infant BCG vaccination being implemented in TB endemic countries (Zwerling et al., 2011). However, despite almost 100 years of experience with this vaccine, several challenges have limited the impact of BCG vaccination policy on the global epidemic. Meta-analysis has shown that BCG vaccination of infants (Relative risk, RR 0.41) and TST-negative children (RR 0.26) offers significant benefit (Mangtani et al., 2014), but BCG vaccination of TST-positive children and older adults is least efficacious (Mangtani et al., 2014). The impact of infant BCG vaccination on pulmonary disease in adolescence and adults, which is responsible for driving the cycle of Mtb transmission in high TB incidence countries, is reduced by the fact that efficacy of infant BCG vaccination is not durable into adulthood (Sterne et al., 1998). Systematic reviews illustrate that, except for a handful of studies in low TB burden settings (Aronson et al., 2004; Barreto et al., 2005; Mangtani et al., 2018), BCG-induced protection wanes to zero by 10–15 years after vaccination (Abubakar et al., 2013; Sterne et al., 1998).

Bacille Calmette Guerin vaccine also has an unfavorable risk profile in people with immune-suppressive conditions, for example congenital immunodeficiencies or HIV infection, due to the BCG bacteremia that follows vaccination even in immune-competent individuals (Trevenen and Pagtakhan, 1982). Severely immune-compromised HIV-infected infants who receive BCG at birth are at risk of developing local, regional and disseminated complications of BCG disease, with incidence up to 992 per 100,000 vaccinated infants and high risk of associated mortality (Hesseling et al., 2009). Recently updated WHO guidelines make provision for BCG vaccination to be considered for HIV-infected children who have been established on antiretroviral therapy, which reduces this risk significantly after immune reconstitution (World Health Organizataion [WHO], 2018a). Future acquisition of HIV infection in adulthood is also a potential safety risk, due to the possibility of reactivation BCG disease (Talbot et al., 1997), but compared to the alternative risk of TB disease the balance of benefit would appear in favor of BCG vaccination in high TB incidence countries.

The limitations of BCG vaccination have encouraged development of novel vaccines designed to boost the BCG-induced immune response and provide additional protection against childhood TB in BCG-primed infants; and novel vaccines designed as a safer and/or more efficacious alternative to replace BCG vaccination in infants. The viral-vectored candidate vaccine MVA85A, a modified Vaccinia Ankara virus expressing antigen 85A, was the first new TB vaccine to enter infant efficacy trials in almost 50 years (Tameris et al., 2013). However, MVA85A boost vaccination of South African infants did not offer additional protection against childhood TB beyond that provided by newborn BCG vaccine, with TB incidence 1,150 per 100,000 in MVA85A recipients vs. 1,390 per 100,000 in controls (Tameris et al., 2013). Two live mycobacterial vaccines intended to replace BCG have also advanced to infant trials in TB endemic countries. VPM1002 is a recombinant urease-deficient BCG expressing listeriolysin, which was designed to be safer and more efficacious than BCG (Nieuwenhuizen et al., 2017). VPM1002 showed an acceptable safety profile in infants and adults in a TB endemic country (Grode et al., 2005); and a safety and immunogenicity trial (NCT02391415) among 416 infants of mothers with and without HIV infection has recently been completed. A larger proof of concept prevention of Mtb infection (POI) efficacy trial is planned to test whether VPM1002 vaccination of newborn infants reduces the risk of subsequent Mtb infection determined by IGRA conversion, compared to standard of care BCG vaccination.

MTBVAC is a live, attenuated clinical strain of Mtb with two independent stable deletions in the virulence genes phoP and fadD26 (Walker et al., 2010). MTBVAC has a similar protein, lipid, and carbohydrate antigen repertoire to virulent Mtb, excluding those antigens regulated by phoP and coded by fadD26; and also contains all the Mtb genes that are in BCG vaccine, plus the *M. bovis* genes that have been deleted from BCG (Arbues et al., 2013; Aguilo et al., 2017). MTBVAC has been tested in an age de-escalation clinical development program among BCG-naïve Swiss adults (Spertini et al., 2015); previously BCG-vaccinated South African adults (Tameris et al., 2019); and recently in BCG-naïve South African infants (Tameris et al., 2019). MTBVAC showed acceptable reactogenicity, induced a durable CD4 T cell response in infants that exceeded that of an equivalent dose of BCG, and demonstrated dose-related IGRA conversion and reversion (Tameris et al., 2019). The encouraging evidence of immunogenicity supports progression of MTBVAC into larger safety and efficacy trials, but also confounds interpretation of tests for Mtb infection, highlighting the need for stringent TB disease endpoint definition. MTBVAC is currently being tested in a larger dose-defining safety and immunogenicity trial (NCT03536117) in preparation for a safety and efficacy prevention of disease (POD) trial in infants.

Planning for infant efficacy trials of live mycobacterial vaccines such as VPM1002 and MTBVAC will be informed by our understanding of infant BCG vaccination as the classical pre-exposure approach. It appears that BCG-mediated protection against TB acts through two component mechanisms: prevention of Mtb infection (POI) and prevention of progression to TB disease (POD) (Roy et al., 2014). Meta-analysis of BCG-vaccinated IGRA-negative children followed for TB disease in 6 studies estimated that overall BCG vaccine efficacy (71%) was the combined result of 27% efficacy against Mtb infection defined by IGRA conversion; and 58% efficacy against progression to TB disease in those children who did become infected (Roy et al., 2014).

Planning for the implementation of novel infant vaccines will also require careful consideration. It is plausible that long-lasting protection against TB from a single infant immunization is not possible and boost vaccination may be required to maintain efficacy. Regular boost vaccination with high coverage may be achievable, but the costs of this strategy to maintain the impact on TB incidence into adolescence and adulthood would increase. Infant vaccinees may also be more difficult to access later in life, potentially lowering coverage; and efficacy may differ, not least because Mtb infection prevalence increases with age. Work is ongoing to estimate the likely effect of these factors on the impact and cost-effectiveness of infant vaccination in different epidemiological and health system contexts.

## Pre-Exposure Approaches to Prevention of Infection (POI) in Adolescents

Two critical questions for new pre-exposure approaches to vaccination of older Mtb-uninfected populations need to be answered: (1) Can vaccination of older children and adolescents offer additional protection against TB disease, beyond that provided by prior infant BCG vaccination; and (2) Is that protection durable into young adulthood, the peak age for TB incidence? Figure 1.

Results from a recent proof-of-concept efficacy trial of BCG revaccination provide indirect evidence relevant to the first question (Nemes et al., 2018a). The POI trial NCT02075203 enrolled 990 previously BCG-vaccinated, HIV-negative, IGRA-negative, otherwise healthy, South African adolescents. Participants were randomized 1:1:1 to receive either BCG revaccination, the protein-subunit candidate vaccine H4 adjuvanted with IC31, or placebo, and were followed 6-monthly for IGRA conversion over 2 years. Neither BCG revaccination nor H4:IC31 protected against the primary endpoint of initial IGRA conversion (Nemes et al., 2018a). However, BCG revaccination showed statistically significant 45% efficacy [95% confidence interval (CI) 6–68%] against the secondary endpoint, IGRA conversion sustained through 3 and 6 months; the efficacy estimate for H4:IC31 (30%) against the same secondary endpoint was not statistically significant (Nemes et al., 2018a). The reduction in sustained IGRA conversion associated with BCG vaccination was a direct consequence of IGRA test reversion (i.e., change from positive to negative), which occurred nearly twice as often (46% vs. 24%) in BCG recipients compared to placebo recipients. Clinical relevance of the POI efficacy signal is suggested by similar 45% efficacy of BCG revaccination against the exploratory endpoint, IGRA conversion >4.0 IU/mL interferon-gamma (Nemes et al., 2018a). IGRA conversion to above the 4.0 IU/mL threshold was associated with much higher risk of progression to TB disease in two diverse populations, i.e., South African infants (40-fold) (Andrews et al., 2017), and Norwegian adults (30-fold) (Winje et al., 2018), compared to those who remained IGRA-negative.

These encouraging proof-of-concept POI results suggest that pre-exposure BCG revaccination of adolescents can protect against sustained Mtb infection. A larger POI trial of BCG revaccination is planned in South Africa to confirm these findings in an expanded study population, including younger children, at additional sites. The BCG REVAX trial will aim to replicate the efficacy signal against sustained Mtb infection with greater precision; assess durability of protection through 4 years after vaccination; and discover and validate immune correlates of vaccine-mediated protection. However, in order to inform policy recommendations for BCG revaccination, the significance of efficacy against POI endpoints (sustained IGRA conversion and conversion >4.0 IU/mL) should be confirmed in trials that test efficacy of vaccination of IGRA-negative populations against progression to TB disease Figure 1.

Two novel vaccine candidates are also being tested for POI efficacy in adolescent and adult populations. Proof of concept POI efficacy trials can be significantly smaller, shorter and cheaper than similar POD trials (Nemes et al., 2019), since IGRA conversion endpoints accrue at rates more than 10-fold that of TB disease in endemic settings where the force of Mtb infection is high (Nemes et al., 2018a). A positive efficacy signal from a POI trial would support advancement of a candidate vaccine into larger and more expensive POD efficacy trials, but this experimental medicine approach to candidate vaccine development is not without drawbacks. First, it is conceivable that those individuals protected against sustained Mtb infection by a vaccine might form part of the 85–90% of infected people who would control that infection by natural immunity and would never progress to TB disease, even in the absence of vaccination (Ellis et al., 2015). In this case, a positive POI efficacy signal might not translate into POD efficacy. Second, it is equally conceivable that a vaccine that would protect against progression to TB disease might not show efficacy against Mtb infection, which occurs by very different pathological mechanisms. In that case, a negative POI efficacy signal might hinder or even halt further development of a potentially efficacious POD vaccine (Nemes et al., 2019). Third, recent studies suggest that heavily Mtb-exposed individuals with negative IGRA or TST have interferon-gamma-independent T and B cell responses to Mtb-specific antigens (ESAT6/CFP10) (Lu et al., 2019). These data indicate that IGRA and TST endpoints do not capture the full spectrum of Mtb-infected individuals, since the presence of these responses suggests previous or current infection with Mtb. The implication for POI trials based only on the IGRA endpoint is that since interferon-gamma-independent conversion and reversion events would not be captured, the true extent of vaccine-induced protection against Mtb infection might be over- or under-estimated. The significance of interferon-gamma-independent T and B cell responses to Mtb-specific antigens for interpretation of clinical trial endpoints may yet become clear from analysis of the planned BCG REVAX trial, and ongoing studies to discover immune correlates of vaccine-mediated protection against Mtb infection (Nemes et al., 2018b) and TB disease (Van Der Meeren et al., 2018).

DAR-901 is an inactivated whole-cell *M. obuense* candidate TB vaccine (von Reyn et al., 2017), which is related to a first-generation multi-dose preparation originally thought to be *M. vaccae* that was shown to have 39% efficacy against bacteriologically confirmed pulmonary TB in previously BCG-vaccinated, HIV-infected Tanzanian adults (von Reyn et al., 2010). DAR-901 is being tested in a POI efficacy trial (NCT02712424) of a three-dose regimen among 650 IGRA-negative Tanzanian adolescents followed for IGRA conversion over 24 months. ID93 + GLA/SE is a polyprotein comprised of the four *M. tuberculosis* antigens Rv1813c, Rv2608, Rv3619c and Rv3620c, formulated with GLA-SE adjuvant, a synthetic toll-like receptor 4 agonist in a stable oil-in-water emulsion (Penn-Nicholson et al., 2018). ID93 + GLA/SE is being tested in a safety and immunogenicity trial (NCT03806686) of a three-dose regimen among 107 previously BCG-vaccinated, IGRA-negative Korean healthcare workers followed for 14 months for IGRA conversion. The results of these trials will inform the design of future POI approaches and improve our understanding of the role of such trials in the development of new candidates.

## Pre-Exposure Approaches to Prevention of Disease (POD) in Adolescents

A key question for TB vaccine stakeholders is whether efficacy of BCG revaccination against sustained Mtb infection (Nemes et al., 2018a), like that observed for primary BCG vaccination, is similarly associated with protection against TB disease in those individuals who do become infected (Ellis et al., 2015). This pivotal question could be answered by a standalone POD trial, or inclusion of a BCG revaccination arm in any POD vaccine efficacy trial that includes IGRA-negative individuals. Such a trial would be large and costly, given that TB incidence rates in IGRA-negative people are approximately one-third that of IGRA-positives (Mahomed et al., 2011a), but this important question could be answered definitively. Funder enthusiasm might be tempered by two large randomized controlled trials that previously tested efficacy of BCG revaccination in Brazil (Rodrigues et al., 2005) and Malawi (Fine and Ponnighaus, 1988) and found no overall reduction in risk of TB disease, but there are important methodological differences to be considered. First, neither trial screened for Mtb infection before vaccination, or tested for acquisition of Mtb infection thereafter, so it is not possible to stratify the results by baseline IGRA status or to evaluate POI efficacy. Second, an earlier trial of primary BCG vaccination at the same Malawi site also did not demonstrate efficacy (Ponnighaus et al., 1992), which raises the possibility that prior mycobacterial exposure might have abolished any potential benefit of BCG in this setting (Black et al., 2002), although the extent to which BCG efficacy is reduced through masking/blocking by prior Mtb infection as compared to non-tuberculous mycobacterial (NTM) exposure has not yet been delineated (Black et al., 2002; Shah et al., 2019). Third, BCG revaccination did show 33% efficacy (95% CI 3–54%) against TB disease in a subgroup of the Brazilian trial among children <11 years of age at the Salvador site (Barreto et al., 2011), a finding that is consistent with protective benefit among younger children with lower prevalence of Mtb infection (and/or NTM exposure) at the time of vaccination. Although some countries previously practised BCG revaccination it has been discontinued from many immunization programs in the context of declining national TB epidemics (Leung et al., 2001). By contrast, the need to discover new vaccines or repurpose old vaccines to prevent TB in the developing countries of Asia and Sub-Saharan Africa remains urgent. Safety and immunogenicity of the live attenuated *M. tuberculosis* candidate vaccine MTBVAC is also being tested in a dose-defining trial (NCT02933281) among IGRA-negative and IGRA-positive South African adults, in preparation for a future pre-exposure POD efficacy trial.

Funding for a pre-exposure efficacy trial of BCG revaccination, VPM1002 or MTBVAC among IGRA-negative adolescents and young adults would compete for limited resources against other candidate TB vaccines and alternative approaches. It remains to be seen whether pre-exposure vaccination of IGRA-negative children and adolescents can provide durable protection against subsequent Mtb infection and TB disease that lasts into adulthood, a factor that would be crucial for impact on the epidemic in high TB transmission settings. The alternative approach, post-exposure vaccination of IGRA-positive individuals, could be tested in shorter, smaller and less costly trials, since TB case endpoints would accrue approximately threefold faster than among IGRA-negatives (Mahomed et al., 2011a).

## Post-Exposure Approaches to Prevention of Disease (POD) in Adults

The primary results of a randomized, double-blind, placebo-controlled efficacy trial (NCT01755598) of the candidate protein-subunit vaccine M72/AS01_*E*_ for prevention of TB disease in individuals who were IGRA-positive at the time of vaccination were recently published (Van Der Meeren et al., 2018). M72 is a fusion protein of two immunogenic Mtb antigens (Mtb32A and Mtb39A) combined with AS01 adjuvant. The trial enrolled 3,573 healthy IGRA-positive, HIV-negative adults aged 18–50 years of age in South Africa, Kenya, and Zambia; most participants (70%) were known to have received prior BCG vaccination. Participants received two injections of either M72/AS01_*E*_ or placebo 1 month apart and were followed for symptomatic TB disease for up to 3 years. Ten participants in the M72/AS01_*E*_ arm and 22 participants in the placebo arm met the primary case definition for bacteriologically confirmed pulmonary TB diagnosed before start of treatment, after mean follow-up of 2.3 years, giving incidence rates of 0.3 and 0.6 cases per 100 person years, respectively. Efficacy of M72/AS01_*E*_ vaccination was 54% (95% CI 3 – 78) against progression to TB disease in this Mtb-infected (IGRA-positive) population (Van Der Meeren et al., 2018). Final results of this trial after 3 years of follow-up showed that incidence rates were unchanged, with 13 cases in the vaccine arm and 26 cases in the placebo arm, giving 50% vaccine efficacy (95% CI 2 – 74) (Tait et al., 2019).

The results of the efficacy trial of M72/AS01_*E*_ are a landmark for TB vaccine development. This is the first positive efficacy signal for an adjuvanted protein-subunit candidate TB vaccine in Mtb-infected individuals, a population for whom BCG is thought to offer little or no protection (Mangtani et al., 2014). The next steps for development of M72/AS01_*E*_ and fast-follower protein-subunit vaccines will be determined by the gaps in our understanding of the public health impact and cost-effectiveness of implementation in different populations and geographic settings. These encouraging results in an IGRA-positive population would need to be confirmed with greater precision in a larger Phase 3 trial that includes a wider age range and sites representative of other epidemiological settings, whilst recognizing that inclusion of populations with lower TB incidence would add to the size and cost of any licensure trial.

Perhaps the most critical knowledge gap is the lack of efficacy data for individuals who are IGRA-negative at the time of vaccination Figure 1. M72/AS01_*E*_ induces a higher M72-specific CD4 T cell response in IGRA-positive compared to IGRA-negative adolescents and adults (Day et al., 2013; Penn-Nicholson et al., 2015). Since Phase 2 studies previously demonstrated safety in IGRA-negative persons (Day et al., 2013; Penn-Nicholson et al., 2015), implementation of an M72/AS01_*E*_ mass vaccination campaign would not need pre-vaccination IGRA testing for safety reasons. However, from the health economics perspective, calculating the cost-effectiveness of community-wide M72/AS01_*E*_ vaccination may hinge on estimates of efficacy in both the Mtb-infected and Mtb-uninfected populations. It is not known whether a two-dose M72/AS01_*E*_ vaccination regimen offers the same level of protection to IGRA-negative and IGRA-positive individuals; whether priming by Mtb infection is a necessary component of the M72/AS01_*E*_ vaccine-induced protection observed in IGRA-positive people; and if so, whether adolescent BCG revaccination could fulfill that priming function prior to M72/AS01_*E*_ vaccination of IGRA-negative adults. The issue of efficacy in IGRA-negative individuals is particularly crucial for age-targeted vaccination of children and adolescents, who have lower prevalence rates of Mtb infection than adults (Mahomed et al., 2006, 2011b), and for mass campaigns in countries such as Kenya (Nduba et al., 2019), Uganda (Mumpe-Mwanja et al., 2015), and China (Gao et al., 2015), where the majority of the adult population is Mtb-uninfected. Efficacy data for pre-exposure vaccination of IGRA-negative populations will likely be crucial to motivate the health system use case, even in high TB burden countries, and any Phase 3 trial should be adequately powered to provide a definitive answer to this question.

The impact of vaccines that are efficacious in pre-exposure versus post-exposure approaches will be influenced by epidemiological setting and vaccine implementation. Determinants of impact include the relative contribution to TB disease burden from recent Mtb transmission versus reactivation, and the prevalence of Mtb infection in the vaccinated population, in particular when considering age-targeted vaccination of children or adolescents in whom Mtb infection prevalence is lower than in adult populations (Harris and White, 2018; Harris et al., 2019; Renardy and Kirschner, 2019). Modeling the impact and cost-effectiveness of M72/AS01_*E*_ vaccination would be the first step when considering feasibility and cost of roll-out. Country-specific models could inform public health stakeholders of the effect of varying the assumptions for efficacy in both the IGRA-negative and IGRA-positive population, efficacy in HIV-infected persons, age of routine vaccination, and the speed and coverage of mass campaigns.

Development of several fast-follower candidate TB vaccines in the clinical pipeline is likely to be accelerated by the positive M72/AS01_*E*_ findings. Protein-subunit vaccines designed for post-exposure approaches include ID93 + GLA/SE; H56:IC31, a fusion protein of the three mycobacterial antigens Ag85B, ESAT-6, and Rv2660c formulated in IC31 adjuvant, which has been dose-optimized for both IGRA-negative and IGRA-positive populations with prior BCG vaccination (Suliman et al., 2018a); and GamTBvac, which consists of a fusion protein of antigens Ag85A, ESAT6 and CFP10 with a dextran-based adjuvant (Tkachuk et al., 2017). Safety and immunogenicity of GamTBvac is being tested in 180 IGRA-negative, previously BCG-vaccinated Russian adults (NCT03878004). Other viral-vectored vaccine candidates in early stage trials include Ad5Ag85A, a serotype-5 adenovirus expressing Ag85A (Jeyanathan et al., 2016), which is being evaluated for intramuscular or aerosol administration (NCT02337270); and TB/FLU-04L, a live-attenuated influenza-A that expresses antigens Ag85A and ESAT-6, which has been evaluated by the intranasal route in IGRA-negative, previously BCG-vaccinated adults (NCT02501421). An inactivated preparation of *M. vaccae*, originally developed for therapeutic use (Yang et al., 2011), has also been tested in a large randomized controlled preventive efficacy trial (NCT01979900) of a six-dose, 2-weekly schedule among 10,000 Chinese adults with strongly positive TST (>15 mm diameter) followed for incident TB disease for at least 2 years; the trial, which ended in 2017, is yet to publish results.

## Prevention of Disease in High-Risk Populations

### Household and Close Contacts

Alternative TB vaccination approaches targeted at high-risk populations, including those with higher IGRA-positive prevalence rates than in surrounding communities, might also be considered for testing in future trials. Vaccination of household or close contacts of a patient with TB is a potential use case for any vaccine that offers protection against progression from Mtb infection to TB disease (Van Der Meeren et al., 2018). Developed, low TB burden countries with established contact-tracing, screening and chemoprophylaxis programs are able to identify IGRA-positive contacts who are offered TB preventive therapy as standard of care (Getahun et al., 2015). New WHO guidelines now recommend that TB preventive therapy should be considered for all close contacts in high TB burden countries, regardless of age or HIV status (World Health Organizataion [WHO], 2018b). The conditional WHO recommendation does not require IGRA testing due to cost and feasibility considerations, since prevalence of IGRA-positivity among close contacts is high (World Health Organizataion [WHO], 2018b). For example, although overall Mtb infection prevalence in the South-East Asia region is estimated at 28% (Cohen et al., 2019), as determined by TST, a study of adult household contacts in India reported 78% prevalence (Dolla et al., 2019). A case could be made for post-exposure vaccination to prevent TB disease among household contacts, but several considerations temper enthusiasm for this approach. First, the observed 50% efficacy of M72/AS01E does not improve on standard of care isoniazid preventive therapy (IPT) (Van Der Meeren et al., 2018), which is estimated to be at least 60% protective (Zenner et al., 2017), and up to 90% protective in fully adherent patients (American Thoracic Society [ATS] and the Centers for Disease Control and Prevention [CDC], 2000). Second, new 1-month and 3-month rifapentine-containing TB preventive therapy regimens with equivalent efficacy (Sterling et al., 2016; Swindells et al., 2019) are increasingly competitive in terms of operational feasibility and patient acceptability, compared to a two-dose vaccination regimen (Van Der Meeren et al., 2018). Third, any theoretical benefit of more durable protection being offered by vaccination compared to TB preventive therapy would depend on reinfection risk and might not be as beneficial in low transmission settings (World Health Organizataion [WHO], 2018b).

Conducting POD efficacy trials of new TB vaccines among household and close contacts may be an attractive solution to the need for diverse country and population inclusion for licensure purposes. For example, although the total burden of TB cases in populous countries like India and China is large (World Health Organizataion [WHO], 2018c), the relatively modest per person TB incidence would make community-based TB vaccine efficacy trials prohibitively large, long, and expensive. By contrast, trials conducted among groups with higher TB incidence, including IGRA-positive or TST-positive persons, or those with known exposure to an infectious TB patient, would allow efficacy trials to be conducted in settings with low rates of community-level Mtb transmission. This consideration applies particularly to vaccine candidates designed for post-exposure approaches in adults (Van Der Meeren et al., 2018), since pre-existing mycobacterial immunity in household contacts might present a challenge for BCG and other live mycobacterial vaccines (Mangtani et al., 2014).

### HIV Infection

HIV-infected persons are at higher risk of progression to TB disease after infection than HIV-uninfected persons (Scriba et al., 2016). Modeling suggests that in South Africa, a high HIV-prevalence setting, the potential population-level impact of a new TB vaccine would be substantially reduced if the vaccine were contraindicated in HIV positive populations (Harris and White, 2018). However, although there is an urgent need for a new effective vaccine that prevents HIV-associated TB disease, a number of safety, immunogenicity and efficacy challenges must be overcome. Live mycobacterial vaccines like BCG, VPM1002, and MTBVAC have an unfavorable risk profile for persons with HIV infection (Hesseling et al., 2008); and therefore inactivated mycobacterial, viral-vectored and protein-subunit candidate vaccines, including M72/AS01_*E*_, might be more appropriate for vaccination of individuals with HIV and other immune-suppressive conditions (Thacher et al., 2014; Kumarasamy et al., 2016, 2018). Studies of vaccination of HIV-infected individuals have reported lower induced immune responses, compared to HIV-uninfected individuals, which were partly restored by antiretroviral therapy (ART) (Thacher et al., 2014; Kumarasamy et al., 2016, 2018). M72/AS01_*E*_ showed an acceptable safety profile and did not affect HIV viral load or CD4 cell counts in HIV-infected Swiss adults, most of whom were receiving ART (Thacher et al., 2014). In a second trial in India, M72/AS01_*E*_ vaccination was well tolerated and induced a polyfunctional M72-specific CD4 T-cell response that persisted through 1 year, and which was higher in patients receiving ART compared to those who were ART-naïve (Kumarasamy et al., 2016, 2018). However, the crux of the problem is that the period of maximal risk for HIV-associated TB is the period prior to immune reconstitution, before starting ART (Lawn et al., 2009). Can vaccination of HIV-infected persons offer protection against TB disease? An inactivated *M. obuense* candidate vaccine, given as a five-dose regimen, previously showed 39% efficacy (95% CI 4–61) against microbiologically confirmed pulmonary TB in previously BCG-vaccinated, HIV-infected Tanzanian adults with CD4 cell count of at least 200 cells/μL (von Reyn et al., 2010), which suggests that vaccination of individuals after diagnosis with HIV infection may be effective. Another key question is whether vaccination prior to acquisition of HIV infection can offer durable protection against TB that is not subverted by subsequent immune-suppression in the period before HIV diagnosis and establishment of ART (Nemes et al., 2018b).

### Approaches to Prevention of Recurrence and Therapeutic Vaccination

Interest in testing novel TB vaccines for therapeutic indications is based on the potential for a vaccine to improve treatment outcomes for both DS- and DR-TB patients (Manjelievskaia et al., 2016); and the opportunity to test novel candidate vaccines in relatively small Prevention of Recurrence (POR) efficacy trials (Ellis et al., 2015). Improved treatment outcomes might include reductions in pulmonary morbidity, TB mortality, treatment default, treatment failure, and post-treatment recurrence, all of which constitute therapeutic indications for vaccination Figure 1.

### Prevention of Recurrence (POR)

The timing of administration relative to the TB treatment course directly affects the potential for vaccination to affect specific outcomes. For example, vaccination at the end of TB treatment, after demonstration of microbiological cure, would only have potential to affect post-treatment outcomes. A post-treatment POR approach would aim to reduce the rate of subsequent recurrence, including both relapse disease in the form of reactivation of quiescent bacilli after apparent microbiological cure; and reinfection disease arising from a new Mtb exposure and infection episode (Marx et al., 2014). POR trials are appealing for demonstration of proof of concept in that the risk of a vaccine-related safety signal, and the potential for adverse impact of TB-related inflammation on vaccine-induced immune responses, are likely lower than if a therapeutic vaccine was administered during treatment. Given that recurrent TB disease occurs at a rate several times that of primary TB disease in the same community (Marx et al., 2014), candidate TB vaccines could be tested for POR efficacy in a much smaller study of TB patients compared to community-based POD efficacy trials, although it must be acknowledged that clinical trials in TB patients are operationally more complex than trials in healthy volunteers. A number of proof of concept POR trials have been designed using this approach, in the hope that a positive POR efficacy signal would accelerate progression into larger POD efficacy trials.

Three protein-subunit candidate TB vaccines have entered clinical trials in TB patients. ID93 + GLA-SE has been tested (NCT02465216) for safety and immunogenicity in a two- or three-dose regimen among 60 HIV-uninfected, South African, drug-sensitive pulmonary TB patients, who were confirmed sputum culture-negative upon completion of treatment, in preparation for larger POR efficacy trials in this population. H56:IC31 is currently being tested (NCT02711735) for safety and efficacy in a two-dose regimen among 900 HIV-uninfected, South African and Tanzanian TB patients, who are vaccinated at the end of successful treatment for drug-sensitive pulmonary TB, when sputum smear-negative, and followed for 12 months for recurrent TB. M72/AS01_*E*_, which was shown to protect IGRA-positive individuals against progression to TB disease (Van Der Meeren et al., 2018), has also been tested in TB patients (NCT01424501), but recruitment in the study ended prematurely because of a high incidence of large injection site redness/swelling reactions (Gillard et al., 2016). A single-dose regimen of the live, recombinant BCG vaccine VPM1002 is also being tested in a multicentre, randomized, placebo-controlled trial (NCT03152903) among 2,000 successfully treated pulmonary TB patients in India, who are followed for 12 months for recurrent TB.

### Therapeutic Vaccination

Therapeutic vaccination during the course of therapy has potential to improve in-treatment outcomes and, provided efficacy is durable, also post-treatment outcomes including reduced risk of recurrent TB disease (POR). However, the possible benefits of early vaccination for pulmonary morbidity, TB mortality, and treatment failure must be considered against possible safety risks, including the Koch phenomenon. For this reason, clinical trials of new therapeutic vaccines might progress the timing of vaccination iteratively from end of treatment toward treatment start, depending on the absence of safety signals, pre-set stop/go immunogenicity criteria, or demonstration of biological effects of vaccination. Potential measures of therapeutic benefit are similar to other host-directed therapies (HDT), and include month 2 sputum culture conversion and proxy measures of bacillary load, pulmonary function tests, and PET/CT extent of lung disease (Wallis and Hafner, 2015). Although the therapeutic goals of vaccination might be similar to those of HDT, the desired immunologic outcomes of therapeutic vaccination might differ from vaccination for POI and POD indications. For example, lung function and disease severity outcomes may be exacerbated by pro-inflammatory innate or adaptive immune responses that might be harmful for therapeutic indications, yet otherwise beneficial for POI and POD indications.

Two inactivated mycobacterial candidate vaccines have been tested as immunotherapeutic adjuncts to TB treatment. *M. vaccae* has been tested among DS- and DR-TB patients in a number of clinical trials in China (Yang et al., 2011; Weng et al., 2016). Meta-analysis of 25 studies involving 2,281 Chinese MDR-TB patients suggested that multi-dose *M. vaccae* during treatment was associated with faster sputum smear conversion and radiographic disease resolution (Weng et al., 2016). A larger meta-analysis of more than 4,000 DS- and DR-TB patients in 54 studies, including six conducted in countries other than China, also reported faster sputum smear conversion and radiographic improvement in *M. vaccae* recipients (Yang et al., 2011). These encouraging data require confirmation in other populations and epidemic settings. A multi-dose regimen of heat-killed *M. indicus pranii* (MIP), previously known as *Mycobacterium w*, has also been tested for therapeutic potential among 890 re-treatment pulmonary TB patients in a randomized, double-blind, placebo-controlled trial in India (Sharma et al., 2017). MIP vacinees showed a modest increase in the rate of sputum culture conversion from 4 weeks onward compared to placebo (67% vs. 57%), but the rate of relapse TB disease was not significantly different (Sharma et al., 2017).

It is important to note that if POR and/or therapeutic vaccine efficacy were demonstrated in combination with current lengthy standard of care TB treatment regimens for DS- or DR-TB, for example by reducing the rate of TB treatment failure, mortality, or recurrence, the vaccine might not be implemented with the aim of achieving the observed reductions in unfavorable treatment outcomes. Rather, the same vaccine might be tested with novel drug combinations toward the ultimate goal of shorter, simpler, and more effective TB treatment regimens.

Prevention of disease vaccines are expected to be agnostic to Mtb drug sensitivity profile, since it is thought there is little or no overlap between vaccine antigens and the proteins and enzymes responsible for TB drug resistance (Manjelievskaia et al., 2016). By contrast, since the severity and extent of TB disease pathology and the resulting inflammatory milieu are directly related to effectiveness of treatment (Chen et al., 2014), the efficacy of therapeutic and POR vaccines might well be regimen-specific, and different for DR- and DS-TB strains. Whilst clinical trial outcomes for DR-TB have improved (Nunn et al., 2019), historical rates of treatment default, treatment failure, and TB mortality have far exceeded rates of post-treatment TB recurrence (Ahuja et al., 2012), since many DR-TB patients have not survived to cure (Ahuja et al., 2012; Aung et al., 2014). The inactivated mycobacterial vaccine candidate RUTI (Nell et al., 2014), a liposomal formulation of fragmented *M. tuberculosis*, is planned to enter a double-blind, placebo-controlled trial of safety and immunogenicity in 27 DR-TB patients after 12 or 16 weeks of successful intensive phase treatment (NCT02711735). By contrast, the pattern of unfavorable treatment outcome for DS-TB is reversed compared to DR-TB, with rates of recurrent TB disease exceeding rates of treatment failure (Gillespie et al., 2014; Jindani et al., 2014; Merle et al., 2014). Thus the potential public health impact of a therapeutic vaccine would appear maximal for DR-TB, whereas impact of a POR vaccine would appear maximal for DS-TB.

## Conclusion

The recent results from an efficacy trial of a novel protein-subunit vaccine, (Van Der Meeren et al., 2018) and of a repurposed indication for licensed BCG vaccine (Nemes et al., 2018a), are major advances for clinical development of both pre- and post-exposure TB vaccination approaches. Two pivotal trials have shown for the first time that both sustained Mtb infection and progression from Mtb infection to TB disease can be prevented by vaccination of IGRA-negative and IGRA-positive populations, respectively (Nemes et al., 2018a; Van Der Meeren et al., 2018). These encouraging results will stimulate funding to expand the clinical pipeline and accelerate empirical testing of new vaccine candidates (Voss et al., 2018), which has been limited by a lack of market incentives to develop new vaccines for a disease that predominantly affects the inhabitants of resource-limited developing countries (Garcia-Basteiro et al., 2016). The recent results also require important decisions that will shape the next generation of clinical trials to address critical knowledge gaps and unequivocally demonstrate the public health value of new TB vaccination strategies for endemic countries. The size and scope of future TB vaccine efficacy trials will require consortia of public and private funders (Matthiessen et al., 2017), concerted political will, and as the centenary of BCG vaccination approaches, an urgent plan of action from the TB vaccine stakeholder community to achieve End TB targets (Uplekar et al., 2015).

## Author Contributions

MH conceived the review and wrote the first draft of the manuscript. All authors contributed to and edited the final manuscript.

## Conflict of Interest

The authors declare that the research was conducted in the absence of any commercial or financial relationships that could be construed as a potential conflict of interest.
